# Hunters who will not report illegal wolf killing: Self-policing or resistance with political overtones?

**DOI:** 10.1007/s13280-021-01588-w

**Published:** 2021-06-17

**Authors:** Ketil Skogen, Erica von Essen, Olve Krange

**Affiliations:** 1 Norwegian Institute for Nature Research NINA, Sognsveien 68, 0855 Oslo, Norway; 2 Department of Social Anthropology, Stockholm University, Stockholm, Sweden

**Keywords:** Enforcement, Hunters, Illegal killing, Resistance, Self-sanctioning, Wolves

## Abstract

Illegal killing of wildlife is challenging conservation efforts worldwide. Ecological research has shown that illegal killing is severely affecting the transboundary Swedish-Norwegian wolf population. A previous study indicated that unwillingness to report illegal killing of wolves among Swedish hunters contains an element of protest against perceived unjust treatment of hunting and hunters but that it could also simply be a reflection of ineffective law enforcement in the backcountry, driving hunters to effect forms of self-policing. Based on a survey of Norwegian hunters, the present research goes one step further. One in five hunters decline to report illegal wolf killings, and unwillingness to report is predicted by lack of trust in environmental institutions and a general anti-elite sentiment. Hunting-related issues and other factors also affect outcomes, but to a lesser degree. We conclude that unwillingness to report is often part of an oppositional stance related not only to wildlife management and conservation, but to contemporary social change in rural areas and perceived societal power relations. It is unlikely that reluctance to report is driven by frustration over inefficient official enforcement. While a political dimension is not always articulated, overlooking it may stoke conflicts and fortify a perception of unjust power relations.

## Introduction

Biologists estimate that illegal killing is a prevalent cause of mortality in Scandinavian large carnivore populations and affects the conservation status of lynx, wolverines, brown bears and wolves in Norway and Sweden. Concerning the transboundary Swedish-Norwegian wolf population, it has been estimated that about half of all mortality was caused by illegal killing from 1991 to 2006 (Liberg et al. 2012) and the number of illegal killings remains high (Liberg et al. 2020). News media regularly report that animals have gone missing in established wolf territories. While there may be several explanations, annual monitoring shows an area in the Swedish counties of Dalarna and Värmland with prime wolf habitat but where wolves seem to disappear before they reproduce. This is known as the "black hole" among biologists and managers, who unanimously blame illegal killings. (SVT 2019a, b). A similar situation seems to exist in adjacent Norwegian counties. Despite a few high-profile police operations and ensuing trials, which have all involved hunters, most instances of illegal wolf killing are never investigated.

Popular maxims among some hunters of ‘we take care of our own’ (Brymer 1991), ‘our own rules guide us’ (Forsyth and Marckese 1993; Enticott 2011) and preferring to ‘shoot, shovel & shut up’ (Liberg et al. 2012) indicate that many poaching instances go unreported (Eliason 1999). The business of hunting collectives, moreover, is sometimes seen by locals and hunting networks as geographically and sometimes morally remote from a central enforcement authority (Holmes 2007). This appears to be a shared logic to many hunters globally, which demonstrate enclaving behavior in relation to law enforcement that infringes on their traditional rights of use (Okihiro 1997; Muth and Bowe 1998). This enclaving modality calls for forms of peer policing and self-censoring rather than involvement from outside officials, amply illustrated in the context of poaching as folk crime (Nurse 2013; White 2016). This much is affirmed by studies on hunters, some of who argue, for example: “there’s another set of system with those people out there in the woods who do these things and agree on what works. That will always be stronger than any regulation and it’s extremely personal.” (Swedish hunter quoted in von Essen 2016, p.153).

Against this background of denunciation of outside legal interference, we set out to investigate Norwegian hunters’ attitudes to reporting hunting-related crimes, self-sanctioning within hunting networks and non-action in the face of various offences. Focusing primarily on hunters' anticipated reactions to illegal killing of wolves, we depart from a recent study by Peterson et al. (2019) in which informal sanctioning among Swedish hunters is connected to the apparent failure of law enforcement to penetrate hunting circles. 27% opted *not* to report illegal wolf killing. While Peterson et al.'s (2019) study is instructive in clarifying preferences for reporting vs non-reporting of hunting violations, it leaves unresolved an important question regarding the origin of hunters’ preference for self-sanctioning (or indeed propensity to do nothing). While Peterson et al. identify a certain aspect of resentment in hunters' responses, it remains unclear how this relates to the interpretation of self-sanctioning as a necessary course of action in the absence of effective official enforcement. Below we clarify these problems and articulate our contribution to filling this gap.

Building on the work of von Essen and Hansen (2018), Peterson et al. make inferences that hunters’ unwillingness to submit to law enforcement is at least partly grounded in wanting a sovereign jurisdiction – guided by autonomous and informal norms and practices, especially in times where the state is seen to be lacking in legitimacy (Brymer 1991; von Essen and Allen 2017). The presence of an alternative normative order therefore raises questions about its function. Peterson et al.’s paper leaves us with two unresolved propositions that can potentially cast the phenomenon of self-policing in different lights: either it is conducted as a matter of convenience and propriety where law practically cannot reach, or it is driven by a more intransigent tendency on the part of some hunters to disregard and even oppose the regulatory regime. While the resistance dimension is recognized, it has not yet been distinguished from self-policing.

Furthermore, Peterson et al.’s identification of opposition as a significant aspect of hunters’ reluctance to report illegal wolf killing was merely tied to hunters’ perception of (unjust) outside power over hunting regulations, and derogatory and unfair treatment of hunting and hunters by “society at large” (Peterson et al. 2019). However, research on attitudes towards wolves and wolf management over the years has shown that skepticism and outright dismissal of the current management (i.e., conservation) regime is usually embedded in a more comprehensive critique of dominant trends not only in land management and conservation, but indeed urban–rural relations and shifting power relations between social segments, often along lines of class (Bisi and Kurki 2008; Borgström 2012; Eriksson 2016; Krange et al. 2017a). Recent research from Norway has demonstrated that acceptance of illegal killing of wolves among hunters is tied to these same factors, measured as anti-elitism, lack of trust in environmental institutions, identification as traditional hunters, level of education and urban vs. rural place of residence (Skogen and Krange 2020). In the general population, acceptance was tied to skepticism towards immigration and the existence of anthropogenic climate change (not measured among hunters) (Krange and Skogen 2020).

In the Nordic context, the depth of hunters and rural residents’ exasperation with their lack of voice in wolf matters, combined with added stressors affecting the countryside and its practices, appear to have precipitated illegal killings (von Essen and Allen 2015; Pohja-Mykrä 2016). Distrust and opposition toward authorities may thus act as both lubricants for wolf killings in particular, and as barriers to hunting peers taking these crimes to the police. The Nordic context of illegal killings of wolves is sometimes cited as unique in terms of its hunting with dog culture, which is a principal interface for wolf attacks (Peltola and Heikkilä 2015). However, the cultural profile of hunters’ resentment toward wolves and wolf conservation policy is paralleled in many parts of the world (refs), where the wolf is seen as an intrusion of government (Wilson 1997)—federal (Eliason 2014) or European (Drenthen 2015; Arts et al. 2018), and a potent symbol for mounting urban–rural tensions (Brownlow 2000) and hunter-and-anti-hunter debates (Simon 2013).

In this paper we explore the assumption that hunters’ propensity to report is affected by similar factors, and not only by perceived hunting-related injustice, or indeed seeing themselves as stand-ins for ineffective law enforcers.

## Research issues

We hypothesize that views on appropriate sanctions—and if sanctions are needed at all—will be embedded in broader “worldviews” (Skogen and Krange 2020) and related to more general forms of opposition or even resistance, not only derived from experiences hunters have *as* hunters but as part of a broader rural counterpublic (von Essen et al. 2015). We also probe whether a propensity to keep authorities at bay and rather handle transgressions internally among hunters reflects a desire to avoid any kind of interference, and not (only or primarily) a reaction to lack of enforcement presence in the backcountry.

## Design

Our paper uses data from a survey of Norwegian hunters, who were asked to state their preferences for various reporting and non-reporting interventions in the event of witnessing a hunting violation. To supplement and develop the findings from Peterson et al (2019) and to assess the role of opposition toward the state and perceived unjust power relations more broadly, we also correlated these answers with hunters’ stated trust in institutions that may be seen as belonging to the field of environmental governance, their inclination towards anti-elitism, and their affinity towards a traditional hunting culture. Within this, we also examine the potential differences between handling things internally and outright supporting illegal killings of wildlife. Thus, we entertain a spectrum of reporting, resolving internally, ignoring or even supporting violations committed by members of one’s hunting team or network of hunting buddies, and show how these concretize related to various hunting-related offenses, e.g., pertaining to different wildlife species.

The first part of the analysis is exploratory as we map Norwegian hunters' anticipated reactions to various hunting-related offences, and most notably shooting a wolf illegally. This provides an indication as to how illegal wolf killing is rated compared to other transgressions.

We then move on to study the relationship between anticipated reactions to illegal wolf killing and other social and cultural factors, including gender, age, level of education and place of residence on an urban–rural scale. We are also interested in whether hunters think they have wolves nearby, will look at how anticipated reactions to illegal wolf killings are influenced by the hunting culture they adhere to, if they think authorities pay heed to hunters' knowledge and experience, and how much they hunt.

The last and crucial step is to include variables that reflect an oppositional stance conceptualized as anti-elitism and degrees of general trust in what we term environmental institutions.

## Materials and methods

All Norwegian hunters are registered in a national public database. From this database a sample of 2400 was drawn. Data collection were done in cooperation with TNS Gallup Norway. After two reminders, postal and SMS, 852 hunters anonymously completed an online questionnaire, resulting in a response rate of 36%. The sample was compared to official numbers regarding big-game and small-game hunters, and regarding hunters from each county (region). The response rate was somewhat higher among big-game hunters (38%) compared to small-game hunters (30%). There was some variation in response rate between counties (from 28 to 41%). Deviation from the hunter register's official numbers regarding age and gender was modest. Nevertheless, the sample is weighted for both these variables in the analyses. All analyses were performed using IBM SPSS Statistics version 26.

### Anticipated reactions to hunting-related offences

We presented the respondents with a selection of hunting-related offences and asked them to state how they anticipated that their hunting team or group of hunting buddies would react. There were three alternative reactions: "We report it to the appropriate authority", "We handle it internally/among ourselves", and "There is no need to do anything". The following transgressions were listed: "Someone leaves weapon in car with bolt in place", "Someone shoots a raptor illegally", "Someone shoots a bear illegally", "Someone shoots a wolf illegally", "Someone hunts while intoxicated" and "Someone deliberately shoots wrong animal".

### Factors potentially influencing reactions to illegal wolf killing

We introduced four staple background variables, namely gender, age, level of education and place of residence on a rural–urban scale. Gender is a simple dichotomous variable, while respondents were requested to place their age in a bracket on a 6-point scale ranging from 15–24 to 65 + . We measured level of education on a 4-point scale, from primary education only to university education of four years or more. Place of residence (self-reported) was scored on a 7-point scale from "small hamlet or scattered settlement" to "Oslo" (The municipality of Oslo with its 700 000 pop. is more than twice the size of the next largest).

Respondents were asked to report whether they believed they had wolves close to where they live, without specifying "close" or performing any control of the likelihood of them actually having wolves nearby. As a factor in forming people's opinions, their beliefs are obviously more salient than a factual situation of which they are unaware. As we are interested in possible effects of the belief that wolves are nearby, the three response options “yes”, “no” and “don’t know” were recoded into a dichotomous variable where “don’t know” was set to equal “no”.

Some variables were directly related to hunting. We asked respondents how many days they hunted last year. Previous research based on qualitative data has indicated that hunters who are sceptical towards wolves are often very dedicated to hunting as a way of life, see hunting as a staple element in local culture, and hold that hunting is an necessary management tool (Krange and Skogen 2011; Fischer et al. 2013; Krange et al. 2017a). We presented respondents with a list of possible motives for hunting, and they were asked to state on a 5-point scale how strongly they agreed. Among these were: "Hunting is very important in my social circle", "I hunt because wildlife populations must be managed", and "I hunt because hunting is an important tradition". With the intention to create an index, we computed Cronbach's Alpha for these three items, arriving at Alpha = 0.64. That is acceptable given the low number of items. We therefore went on to construct the index as a mean score and named it "Tradition and stewardship".

We further asked respondents about their impression of how much weight decision-makers attach to hunters' knowledge and experience. Answers were scored on a 5-point scale from "no weight at all" to "very much weight". We constructed an index consisting of the mean score on two items from an instrument comprising a list of actors who may or may not take hunters' knowledge into account: "Parliament and government" and "The Environment Agency". Cronbach's Alpha is meaningless when there are only two items, but a simple correlation test yields a Pearson's r = 0.66, which shows that the two items are quite closely related.

In order to operationalize anti-elitism, we selected some items from an instrument intended to tap what has been termed "political alienation" (Eriksson 2016) as well as anti-elitism. The selected items were: "The elites (top people in politics, business and public administration) determine how society develops over the heads of ordinary people", "Politicians are mostly concerned with securing their own positions", "Experts without practical experience decide too much in this country", "Ordinary people are more honest than politicians" and "Sound common sense is better than formal education". Responses were scored on a 5-point scale ranging from "strongly disagree" to "strongly agree". The items yield a Cronbach's Alpha at 0.84, indicating strong internal consistency, and we constructed the index as a mean score.

Finally, we introduced variables we have often used in previous research, and these always play an important role in predicting attitudes towards wolves as well as other environmental issues, namely confidence in environmental institutions. This ties directly to the legitimacy of these institutions. We use the term broadly, because the "environmental segment" including government institutions, scientists, and mainstream NGOs is often seen as a coherent whole (or as a monolithic conglomerate, depending on the perspective of the beholder) (Skogen and Thrane 2007; Krange et al. 2019). Given that these collective actors adhere to shared discourses about nature and also have extensive interaction, this understanding is clearly not unfounded. To construct an index, we included the Ministry of Climate and Environment, the Environment Agency, the Nature Inspectorate (SNO, essentially a national ranger service), The Norwegian Society for the Conservation of Nature (large conservation NGO), wildlife biologists, climate scientists and police units that investigate wildlife crime. Respondents were asked to indicate their level of trust in these actors on a 5-point scale from "very high trust" to "no trust at all" when it comes to "climate and environment issues", i.e. not directly related to either hunting, wolves, wildlife, or even conservation. The items have a Cronbach's Alpha of 0.87, indicating high internal consistency.

## Results

### Anticipated reactions to hunting-related offences

**Fig. 1:**
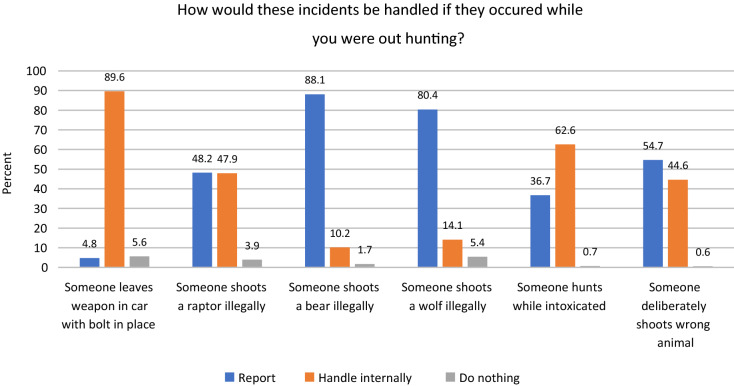
How hunters think hunting-related offences would be handled. N = 851

How does illegal wolf killing compare to other hunting-related offences? In Fig. 1, these other offences are displayed along with killing a wolf illegally, and we can see the percentage of respondents in each category: "Report", "handle internally" and "do nothing".

Shooting a bear illegally is the offence that the largest percentage of hunters would report but shooting a wolf would also be reported by a large majority. However, there is a difference of almost eight percentage points between the two. Shooting a raptor illegally would be reported by considerably fewer, as would shooting wrong animal deliberately, hunting while intoxicated, and—in particular—leaving gun in car. The latter is something that could happen by accident, and there could be mitigating circumstances (nobody around, not loaded, etc.). However, we can see that leaving a gun in the car would normally be handled internally (clearly the offence where the largest percentage opted for this alternative), meaning that it would not just be overlooked. The "handle internally" option is also endorsed by sizeable groups concerning hunting while intoxicated, shooting wrong animal, and shooting a raptor illegally. These percentages are much smaller for shooting a bear or wolf illegally, but we notice that there is a difference between the two here as well: While about 10% would handle shooting a bear internally, a little over 14% would do the same if it was a wolf. More than three times as many hunters would "do nothing" if a wolf were killed, compared to a situation with an illegally killed bear. Taken together, the "do nothing" and "handle internally" categories account for 19.5% of hunters if a wolf is killed, and 11.9% if it is a bear. This is a notable difference and supports the observations in previous research that wolves are more controversial than other large carnivore (Skogen and Krange 2020).

### Bivariate models

Our main objective in this article is to observe the differences between the categories "report", "handle internally" and "do nothing" when shooting a wolf illegally is the offence in question, related to the background variables presented above. We start this endeavor using one-way ANOVA, and the variable we focus on throughout—anticipated reaction if somebody shoots a wolf illegally—is assigned the role of independent variable. This enables us to present the results in a simple manner: Table 1 shows the mean scores on the background variables for each of the categories of anticipated reactions. What we observe here are the bivariate relationships, providing an instructive overview.

**Table 1:** Bivariate relationships between anticipated reactions and background variables. One-way ANOVA

	Rural–urban	Education	Wolves nearby	Number of days hunting	Hunting motive: Tradition and stewardship	Authorities heed hunters’ knowledge	Anti-elitism	Trust in environmental institutions
Report	3.7	2.7	0.36	14.9	3.5	1.9	3.7	3.2
Handle internally	3.5	2.4	0.48	17.8	3.7	1.6	4.05	2.5
Do nothing	2.9	2.2	0.55	23.5	4.0	1.4	4.18	2.3
F	4.72	11.26	5.67	7.68	7.05	27.23	14.87	47.6
*P*	< 0.01	< 0.001	< 0.01	< 0.01	< 0.01	< 0.001	< 0.001	< 0.001

First, we determined that the standard background variables gender and age were not associated with hunters' opinions on likely courses of action. This may be surprising given what we know about these variables and attitudes towards wolves in the general population (Krange et al. 2017b) but (1) our sample consists of hunters only, and (2) attitudes towards wolves are not the same as views on how illegal killing should be dealt with. As there are no effects, we do not include these variables in the analyses henceforth.

As we can see from Table 1, the group of respondents who anticipate that illegal killing would be reported to the authorities have a higher mean score on the rural–urban scale (meaning they tend to be less rural) than those who would do nothing, and also compared to those who would handle things internally. The *F*-value for the model is significant at the *p* < 0.001 level, but we need post hoc testing (we used Hochberg's GT) to tell us where the significant differences lie. The “report” category is significantly different from “do nothing”, but "handle internally" is not significantly different from either of the other two (at the *p* < 0.05 level). This means that there is a significant tendency that less urban hunters are more inclined to do nothing than their more urban peers, and conversely less inclined to report. However, place of residence does not affect affinity for taking care of such transgressions among hunters without involving authorities.

In the next column in Table 1, we see that those who are inclined to report tend to have more education than those who are not. In this case the post hoc testing reveals that the “report” category is significantly different from both the others. These, however, do not differ from each other.

The belief that there are wolves nearby was associated with hunters' views on courses of action: Perceiving wolves as close is more common among those who will do nothing or handle things internally compared to those who will report. Post hoc testing shows that the “report” category is significantly different form both the two others, but, again, these two are not significantly different from each other.

Hunters who anticipate that illegal killing would be reported were hunting fewer days last year than those who will do nothing. The significant difference only exists between the categories "report" and "do nothing". This, then, is another variable where only the category implying indifference or support for illegal killing is significantly related to the dependent variable, whereas the one expressing propensity to act, but without outside involvement, is not.

Those who do not want to report score higher on the index for hunting motive labeled “tradition and stewardship”, compared to those who will do nothing. However, the only significant difference is found between the two extremes, none of which are significantly different from "handle internally".

Hunters who are inclined to report score higher on the index intended to measure how much weight authorities are thought to attach to hunters’ experience and knowledge, compared to the two other groups. The latter are not significantly different from each other.

Those who anticipate that they would do nothing or would handle things internally score higher on the anti-elitism index compared to those who would report. The first two categories are not significantly different from each other, but both are significantly different from "report".

Those who are inclined to report tend to have a higher trust in environmental institutions than those who will not report. Post hoc testing tells us that there is no significant difference between those who will handle things internally and those who will do nothing.

To sum up, we see that the mean scores on the variables *place of residence on the rural–urban scale*, *days hunting*, and *hunting motive,* are only significantly different between the categories “report” and “do nothing”. The category “handle internally” does not yield a mean score on these variables that differs significantly from the other two. The rest of our variables, *education*, *perception of having wolves nearby, perception of authorities’ respect for hunters’ knowledge, anti-elitism,* and *trust in environmental institutions,* have mean scores that are significantly different between both “do nothing” and “handle internally” on the one side, and “report” on the other. No variable presents means that are significantly different between “do nothing” and “handle internally”.

A comparison of the *F*-values in Table 1 shows that the variables that are neither “material” (place of residence, wolves nearby) or related to hunting in general (days hunting, hunting motive), but rather comprise dimensions of scepticism towards authorities and “elites” and an oppositional stance (generally and related to hunting issues such as the impact of hunters’ knowledge on regulations etc.) have a more considerable impact on the *F*-values. This comparison is not a stringent statistical one, but a rough overview. We maintain that it can be interpreted as a stronger relationship between these latter factors and anticipated reactions to illegal wolf killings.

### Multivariate model

In order to investigate how the variables work in concert, we performed a multinominal logistic regression. The results are shown in Table 2. We used "report" as our reference category, meaning that the other two categories are compared to it.

**Table 2:** Multinominal logistic regression: Hunters’ propensity to “handle internally” or “do nothing” compared to “report”

	SE		*P*	OR
	B	B		
Handle internally				
Intercept	2.029	1.221	0.096	
Rural–urban	0.101	0.079	0.198	1.107
Education	− 0.284	0.162	0.080	0.753
Wolves nearby	0.239	0.238	0.316	1.270
Days hunting	− 0.004	0.007	0.606	0.996
Hunting motive tradition stewardship	− 0.029	0.135	0.831	0.971
Authorities heed hunters’ knowledge	− 0.411	0.234	0.079	0.663
Anti-elite	− 0.013	0.172	0.939	0.987
Trust environmental institutions	− 0.856	0.173	0.000	0.425
Do nothing				
Intercept	2.095	1.976	0.289	
Rural–urban	0.055	0.132	0.679	1.056
Education	− 0.561	0.272	0.039	0.571
Wolves nearby	0.595	0.381	0.118	1.813
Days hunting	0.001	0.010	0.940	1.001
Hunting motive tradition stewardship	0.074	0.227	0.743	1.077
Authorities heed hunters’ knowledge	− 0.973	0.403	0.016	0.378
Anti-elite	− 0.103	0.288	0.721	0.902
Trust environmental institutions	− 0.786	0.282	0.005	0.455

Our first observation is that many relationships that were significant in the ANOVA analysis, although not very strong judged from the F-values, are not significant in the multivariate regression model. The point here is of course not to compare results from statistical procedures based on different logics, but to see how effects respond when variables are allowed to interact.

If we look first at the "do nothing" alternative as compared to "report", we observe that three variables yield an odds ratio (OR) significant at least at the 0.05 level. These are level of education, trust in environmental institutions, and to what degree authorities are perceived to take hunters' knowledge into account. This means that hunters who have less education, less trust in environmental institutions, and who do not think authorities pay much heed to hunters' knowledge are more inclined to do nothing if a wolf is killed illegally. The ORs show us that the effect of trust is stronger than that of education and opinions on the authorities’ acknowledgement of hunters' experience.

When comparing “report” and “handle internally”, we see that only the trust variable yields a significant effect on OR. Those who have less trust in environmental institutions are more inclined to handle incidents among themselves.

The ANOVA results showed that here were significant effects of all the background variables we tested (except age and gender). Of course, these effects do not evaporate even though they do not affect OR in a multivariate model where all variables interact. They are, however, in a sense swamped by the stronger and more direct effect of trust, to which they are also related. The same goes for the persistent yet somewhat weaker effects (related to the “do nothing” category) of level of education and beliefs about authorities respect for hunters’ knowledge: They are connected to the dependent variable in more direct ways so that they absorb the more opaque relationships.

## Discussion

In line with what has previously been observed in studies of environmental attitudes, including attitudes towards wolves (Skogen and Thrane 2007; Krange et al. 2017a) but also climate change (Krange and Skogen 2019) trust in the institutions and collective actors that share and manage a hegemonic discourse in the field of environmental politics is a defining part of what we for the sake of brevity may term comprehensive “worldviews” (Skogen and Krange 2020). In this case, a low level of trust is a predictor of seeing illegal wolf killing as something that should not be reported. We have seen that anti-elitism also plays a part, as does level of education, and the notion that authorities do not take hunters’ knowledge into account.

The latter variable is clearly related to hunting, but also connects to the two others in its relation to struggle over legitimate knowledge, a core component in cultural resistance with political overtones (Krange et al. 2017a). Factors that do not in and of themselves connote a broader scepticism towards authorities and “elites” do indeed also play a part. Level of education cannot be taken to equal such scepticism, but research over many years has demonstrated that social class (for which level of education can be taken as a proxy) is a core element in struggles over the dominant status of academic knowledge.

Factors of a more material nature, such as place of residence, wolves in the vicinity and number of hunting days, do play a part but are swamped by trust and level of education in a multivariate model. The same is the case with traditional hunting motivation: There are significant differences between our categories in the bivariate ANOVA, but effects disappear in the multivariate model, indicating that this too is channeled through trust and level of education.

It may well be that hunters’ self-policing or non-reaction to offenses in some cases reflect the observation that law enforcement is simply not effective (Gavin et al. 2010; Bunnefeld et al. 2013). Accordingly, hunters need not bother with involving the police, whether or not they will impose reactions of their own. We anticipated that the relative role this belief holds may be partly elucidated when distinguishing self-sanctioning from non-action. This is obviously also a simplification, as a mixture of motives can co-exist within any group or even within one individual. However, in relative terms, self-sanctioning signals a desire for correction and proactivity, while non-action signals, if anything, that one does not consider the offense to be a crime worthy of punishment. And indeed, “do nothing” is a more marked contrast to “report” than is “handle internally”. To do nothing could be seen as a more direct rebuttal of the legitimacy of authorities (Ballesteros et al. 2020). Still, the overall picture is that the two “non-report” categories are not significantly different from each other, meaning that they are essentially associated with the same factors in our models, albeit to different degrees.

We must also consider the social context in which different views on appropriate reactions play out. We problematize the term “hunting team” in this context to nuance the perhaps generalizing way in which Peterson et al. (2019) deploy it for their inquiry. Such nuancing is important, since hunting teams are not static but concretize in multiple communities of practice differentiated by type of game, time of year, use of dogs, hunting method and more (von Essen et al. 2015). Much game in Scandinavia is currently *not* hunted by teams—small-game is typically hunted alone, as is big game like wild boar and wild reindeer. For this reason, it may be tenuous to talk about a hunting team as exerting a sanctioning influence on offenses in any direct way. Furthermore, other hunts are conducted in loose, sporadic team configurations. Many are close-knit and have continuity, even inter-generationally, but there is a growing demographic of hunters who mostly join up for seasonal moose or deer hunting, and who do not have much in the way of lasting affinities with their hunting peers or attachment to the community (Gunnarsdotter 2005; Hansen et al. 2012).

To further complicate things, Scandinavian moose hunting teams are highly differentiated in terms of roles and formalized in terms of responsibilities and moral codes—including any sanctioning for wrongdoings. It may be for this reason that hunters have declared moose hunting teams a paragon of ethical conduct, holding themselves to a higher standard than many other hunts, where corners may be cut (von Essen 2016). There is also an elaborate monitoring system on moose and moose harvest that provides transparency and trust in bag limits (Singh et al. 2014). This potentially means that the normative order found in moose hunting teams is not representative of hunting teams broadly, and even less so of looser networks of hunters. All in all, there can be no such thing as a consistent informal normative order among hunters, and not even in hunting teams.

The function of an informal self-sanctioning system among hunters may be conceptualized differently depending on whether one departs from the phenomenological perspective of hunters, as compared to an analytic reading. We have conducted a questionnaire-based survey, and do not have access to the self-understanding of hunters in reporting or non-reporting transgressions. The best we can do is correlate these preferences for actions with various other variables. In connection with this, it is also pertinent to observe that the motives of hunters in shunning outside regulatory interference may be opaque also to themselves, as von Essen (2016) notes. But the cumulative effect of one out of five hunters choosing to distance themselves from the law, in combination with holding skeptical attitudes toward the state and “elites”, may be analytically parsed as a pattern of resistance.

Seen in connection with the effects of rural place of residence, limited formal education, and association with traditional hunting motives and a considerable hunting activity, we discern a pattern where reluctance to report illegal killing is part of a form of cultural resistance (Krange and Skogen 2011). By this we mean a defiance of hegemonic discourses, often subdued so that Scott's term "infrapolitics" (Scott 1992) seems useful: the political dimension is there if we look closely, but it is not obvious on the surface. Similarly, the observation of a defiant "counterpublic" in qualitative studies (Holmes 2007; von Essen et al. 2015; Carter et al. 2017) seems to receive support here, because expressing (some degree of) acceptance for illegal killing (not wanting to report it) in the context of distrust for environmental institutions and anti-elitism points in the same direction, as does the notion that hunters’ knowledge (as opposed to academic knowledge) is not taken seriously. Previous research has shown that hunters themselves tend to see illegal wolf hunting as a form of protest and seem to be aware of the existence among hunters of such a counterpublic, regardless of whether they see themselves as part of it (Skogen and Krange 2020).

Protection of large carnivores is perceived by some social groups as an expression of a changing land use regime, seen as threatening rural economic activities and traditional rural lifestyles (Sjölander-Lindqvist et al. 2015; Krange et al. 2017a). The back-curtain is economic decline, leading to depopulation and dismantling of private and public services in rural areas. This occurs in a time when a conservation ethos has achieved a central position in the public discourse and manifests itself in practical land management. Accordingly, opposing protection of large carnivores may be seen as defending the rural economy and rural culture against harmful outside forces.

The strong effect of lacking trust in environmental institutions tells us that government institutions and the discourse on conservation they are associated with are facing a legitimacy challenge. This seems to be related to a deeper sense of disenfranchisement among some hunters, leading to the packaging of illegal wolf hunting as a form of—more or less—legitimate resistance against power that not only controls wolf management, but is also seen as underlying unfair urban–rural relations and advancing the interests of social segments branded as "elites". The links to perspectives that underpin much of the current populism literature are evident (Mamonova and Franquesa 2020).

We see few traces of support for self-regulatory efforts intended to replace inefficient official enforcement. Indeed, sanctions among peers would not exclude simultaneous activation of the official apparatus, and those hunters who foresee such a combination would in all likelihood opt for “report” in our survey. The propensity to opt for “handle internally” and not report, is related to the same factors (prominently including lack of trust in institutions) as “do nothing”, albeit to a somewhat lesser degree.

Our findings indicate that treating all breaches of wildlife law as criminal conduct that should be pursued by traditional means (policing and prosecution) will probably not be effective in the long term. This appears to be confirmed by emerging studies in the natural resource management context more broadly (Ballesteros et al. 2020). Indeed, it is likely that increased surveillance and use of force will stoke conflicts on the ground, as such actions probably will be seen as a confirmation of the understanding of power relations that the current resistance (the infrapolitics) is already embedded within. In concert with a large body of research on conflicts over wildlife management and conservation, our findings also indicate that efforts addressing illegal wolf killing, or wolf management for that matter, in isolation from larger socio-political struggles—well outside the control of wildlife management agencies and law enforcement—will face an uphill battle. Comprehensive strategies must address rural development and societal power structures, openly acknowledging the political nature of conflicts such as those culminating in illegal killing of large carnivores.
